# Comparison of the Agonist/Antagonist Tensional Balance of the Knee between Two Isokinetic Positions: A Pilot Study on a Sample of High-Level Competitive Soccer Players

**DOI:** 10.3390/ijerph19095397

**Published:** 2022-04-28

**Authors:** Jorge García-Pastor, Ildefonso Alvear-Ordenes, Diego Arias-Giráldez, María Mercedes Reguera-García, Beatriz Alonso-Cortés

**Affiliations:** 1 Centro de Fisioterapia y Readaptación García-Pastor, 33010 Oviedo, Spain; fisioterapiagarciapastor@gmail.com; 2 Laboratory of Applied Physiology (FISAP), University of León, 24404 Ponferrada, Spain; balof@unileon.es; 3 Institute of Biomedicine (IBIOMED), University of León, 24071 León, Spain; 4 Centro Deportivo One Sport Giráldez, 24402 Ponferrada, Spain; diegui14@hotmail.com; 5 SALBIS Research Group, Faculty of Health Sciences, Campus de Ponferrada, University of León, 24401 Ponferrada, Spain; mmregg@unileon.es; 6 Department of Nursing and Physiotherapy, University of León, 24071 León, Spain

**Keywords:** knee joint, hamstring muscles, quadriceps muscles, isokinetic testing, unified reclined position

## Abstract

Isokinetic knee dynamometry evolves towards more precise techniques, such as the calculation of the functional ratio. This study evaluated the influence of an intermediate hip position called the unified reclined position (URP) compared to the classic sitting position, (CSP) on hamstring eccentric PT values (H_exc30_) and conventional (H_con60_/Q_con60_) and functional (H_exc30_/Q_con60_) ratios. Twenty Spanish high-level competitive soccer players (20.4 ± 4.44 years) were evaluated in CSP and in URP. The hip angle in URP (44°) was determined with a passive extensibility test (quadriceps and hamstrings), looking for an agonist/antagonist tension balance. The following were performed: three repetitions (60°/s) and five repetitions (240°/s) in concentric quadriceps and hamstrings mode; and three repetitions (30°/s) in concentric and eccentric for the hamstrings. At 30°/s, the CSP presents higher values of maximal eccentric hamstring strength than URP, (Dom + N-Dom leg (Nm): CSP = 148.3 ± 19.5 vs. URP 143.5 ± 23.2); *p* = 0.086 (n.s.). The conventional relationship did not show data justifying the preference for URP over CSP (*p* = 0.86 (n.s.)). However, although the functional index did not show significant values (*p* = 0.97 (n.s.), it did show a greater number of subjects with imbalances measured in URP (five in URP vs. two in CSP). An assessment angle of the hip closer to sports reality seems to favor the use of the URP as a complementary method to the CSP. These data stimulate new studies using URP together with the classic protocol.

## 1. Introduction

Isokinetic dynamometry is a tool commonly used, among other purposes, to assess the physical condition and effectiveness of the rehabilitation process [1,2], or to identify injury risk factors in athletes, including soccer players [3]. Muscle imbalances can thus be observed through the ratios obtained, bilaterally or unilaterally. Among the body areas most studied by the isokinetic methodology is the knee, where muscle imbalances play a key role, contributing to a high number of injuries in the hamstring and the anterior cruciate ligament (ACL) [3,4,5,6].

One of the most widespread study modalities of these imbalances is the conventional ratio, which results from the quotient between the hamstring (H) peak torque (PT) and the quadriceps (Q) PT according to the concentric (CON) modality, both at a speed of 60°/s (H_CON60_/Q_CON60_) [1,7,8,9]. A calculation of the conventional ratio below the normality reference value of 60% indicates a marked weakness of the hamstring in relation to the quadriceps, which is, in turn, indicative of the risk of injury to the hamstring and/or ACL [10,11]. However, this evaluation modality is being questioned because it indicates that the muscles that functionally perform contrary actions and in different contraction modalities should not be evaluated in the same way [12,13,14].

There is great interest in knowing the data on the eccentric PT of the hamstring muscles since it is in this phase that the main non-contact injuries occur [15,16]. In addition to this fact, the ACL breaks, among other reasons, as a consequence of insufficient eccentric strength of this musculature of the posterior region of the thigh [15,16]. The “dynamic control ratio” (DCR), also known as the functional ratio (eccentric hamstrings to concentric quadriceps) [12,13], was developed to solve these problems. Seeking to reproduce the movements with greater precision, Croisier et al. [14,17] evaluated the hamstrings eccentrically at a speed of 30°/s and the quadriceps in a concentric way at 240°/s (H_ECC30_/Q_CON240_). Furthermore, the eccentric isokinetic tests at a slow angular velocity allow an evaluation of the maximum hamstring strength [14,17]. Croisier’s choice, of an eccentric hamstring speed of 30°/s, was due to the fact that high eccentric speeds, could lead to inaccurate results. In addition, the exercise is more easily perceived and performed at low speeds [14,17].

The modality developed by Croisier [14,17], known as the functional ratio, was used by other authors such as Ayala et al. [18] to determine the existence of the risk of injury when the values are below 98%. However, although it is claimed that this is the best predictor of injuries [19], it is also known that there are still 5% of injuries that cannot be detected [20].

**Figure 1:**
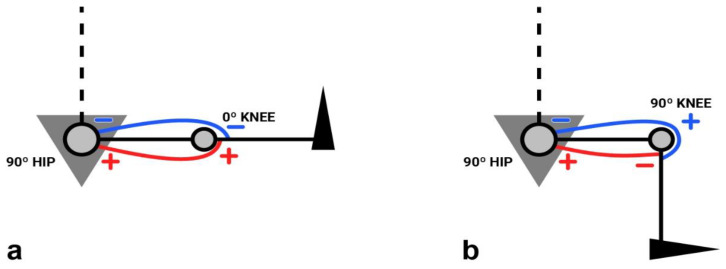
Diagram showing the difference in pretension between the quadriceps and hamstring muscles when the knee is in extension (**a**) and flexion (**b**). The “+” and “−” signs, which can be seen in both the areas of origin and insertion of the muscles, represent high pretension (+) and low pretension (−). The red lines symbolize the hamstring muscle, and the blue lines symbolize the quadriceps muscle.

The isokinetic knee assessment is usually performed in the seated position [14,17,21]. Nevertheless, for the thigh musculature, the classic sitting position (CSP) cannot be considered neutral [18], since with a hip flexion close to 90°, the hamstrings present a greater pretension than the quadriceps at the proximal level; that is, near the insertion area in the pelvis. To this situation, we must add the increase in distal hamstring pretension (close to the knee insertion area). This maximum pretension occurs both in the initial phase of concentric knee flexion and in the final phase of eccentric knee extension, i.e., when the joint is stretched. In this position, with the hip flexed (90°) and the knee extended (0°), the hamstrings are in the maximum stretch. However, with both hip (90°) and knee flexed (90°), the stretching degree of the quadriceps at the beginning of its contraction is much lower (Figure 1). This anatomical-physiological factor (length-tension difference between the muscles involved) may contribute to achieving a greater PT of the hamstrings, higher than that generated without pretension. However, the quadriceps is not benefited to the same extent, so the hamstring/quadriceps (H/Q) ratios could also be altered.

**Figure 2:**
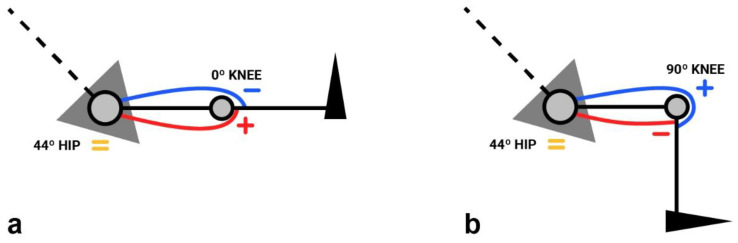
Diagram showing the difference in pretension between the quadriceps and hamstring muscles distally (knee) and equal tension proximally (hip) when the knee is in extension (**a**) and flexion (**b**). The “+” and “−” signs can be seen only in the muscle insertion zones. The “=” sign can be seen in the proximal insertion zone of the muscles. “+” means high pretension, “−” low pretension and “=” equal pretension. The red lines symbolize the hamstring muscle, and the blue lines symbolize the quadriceps muscle.

This situation stimulates the search for a position that considers the influence of the hip position on the muscular component (Figure 2). In this sense, the most recent studies seem to indicate that the classic sitting position (CSP) allows the development of a higher knee flexor PT than any other position tested [22,23]. It also seems clear that as hip flexion increases, the hamstring PT also rises [24]. Nevertheless, the quadriceps does not appear to be altered with the change in a position [23,24]. As the recordings of one of the affected muscles vary, the ratio is also altered by the change in a position [20,23]. However, to date, no study has considered the elasticity and/or stiffness of the musculature involved in the choice of this position.

A unified reclined position (URP, see Section 2.3), whose choice takes into account the elasticity/stiffness of the muscles involved, would allow recording the PT values obtained under equal conditions of muscle-tendon units tension and lengthening. This change in the evaluation position will modify the unilateral registers, i.e., the H/Q ratio, but that should not be the case in bilateral ratios (H/H) or asymmetries.

The objective of this study is to assess the possible differences caused by the change of position in isokinetic knee tests in URP and CSP positions regarding knee strength results, hamstring eccentric PT, and conventional and functional ratios.

## 2. Materials and Methods

### 2.1. Experimental Approach to the Problem

**Figure 3:**
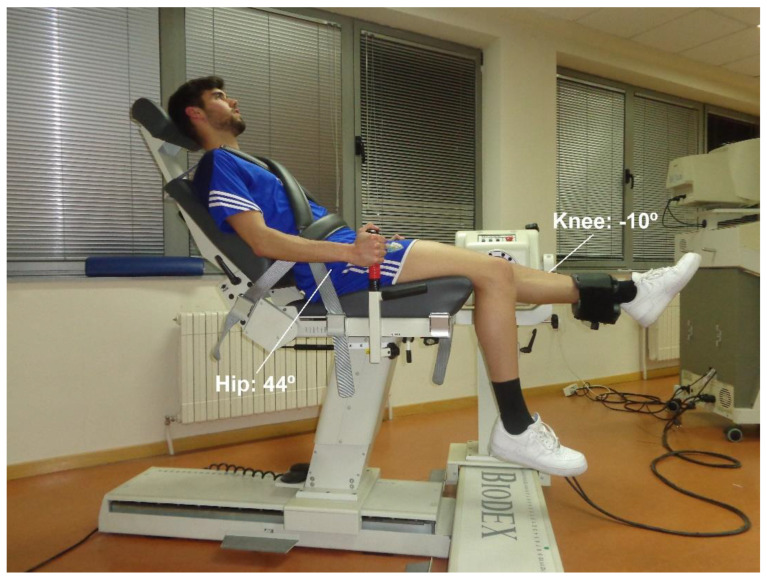
One of the sample subjects in the unified reclined position, at a 44° hip flexion and −10° knee extension.

The research described in this paper is based on an observational study. Two protocols for assessing the isokinetic force in knee flexion-extension were compared, which differed in their hip position: the CSP (90° hip angle), and the URP hip angle (URP_ANGLE_) at 44° (Figure 3), obtained from a formula based on the elasticity of the quadriceps and hamstring (see Procedures). In each test, both legs were evaluated in each hip position, and at three different speeds as recommended by Croisier et al. [14,17]. To optimize time, no randomization was applied, strictly speaking, in the order of evaluation of the subjects. In return, before performing the tests, a random evaluation sequence was determined, which established the start of the protocol (CSP/right leg, CSP/left leg, URP/right leg, URP/left leg) for the first 10 subjects evaluated. The next 10 subjects followed the opposite sequence. In turn, half of each group of ten began the test with the right leg and the other half with the left. In this way, the same number of subjects began the isokinetic test with one of the four possible alternatives. This protocol was created on a right/left leg basis and not on a dominant/non-dominant leg basis, to be agile and fast in obtaining isokinetic results. In this way, less time is wasted on modifying the seat of the isokinetic machine (goniometric measurement) with each subject and execution position. This division into groups prevented muscle overload from altering the results. What could not be ascertained is whether fatigue after the execution of one leg might influence the results during the execution of the other leg.

### 2.2. Subjects

The study sample consisted of 20 Spanish high-level competitive soccer players belonging to the affiliate team of a soccer club of the Spanish second league. All the subjects were informed of the objectives, of the protocols to follow, and signed an informed consent before participating in the study. In the case of participants who were under 18 years of age, a consent form was signed by their parents or legal guardians. The experimental design was approved by the Ethics Review Board of the University of León (ÉTICA-ULE-013-2015) and followed all the national and international regulations (ICH Guidelines and Good Clinical Practices Guidelines).

The sample presented a mean and standard deviation age of 20.4 ± 4.44 years old, a body height of 176.5 ± 6.15 cm and a body mass of 71.54 ± 6.78 kg. Regarding the dominance of the lower limb, 15 subjects were right-footed and five were left-footed. Each subject freely determined which was their dominant leg. For some it was the leg they use to hit the ball, while for others it was the leg they use to jump.

The eligibility criteria did not consider the subjects’ age or dominant leg. Furthermore, none of the subjects had suffered a muscle (hamstring or quadriceps) or knee injury over the past year [23]. The evaluations were conducted in the middle of the season, and the only exclusion was due to a lower limb injury in one subject, which prevented him from performing the evaluations.

### 2.3. Procedures

**Figure 4:**
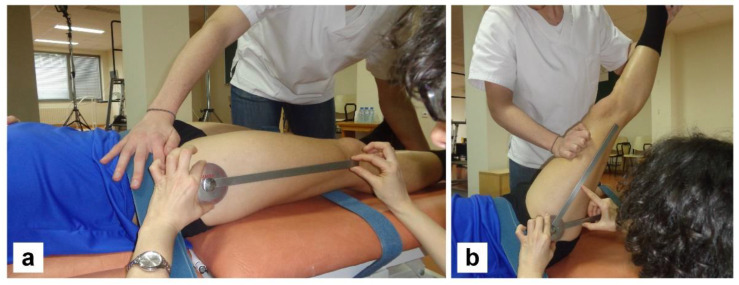
Start (**a**) and end (**b**) positions of the straight leg raised (SLR) test, performed in the supine position.

**Figure 5:**
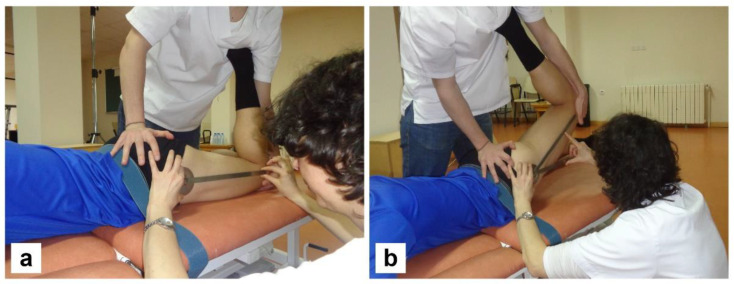
Start (**a**) and end (**b**) positions of the quadriceps extensibility test, performed in the prone position.

The subjects made two visits to the laboratory. During the first visit, measurements of the passive elasticity of the hamstring and quadriceps muscles were taken in both legs. The hamstring musculature was analyzed by means of the straight leg raise (SLR) test [18], performed in the supine position (Figure 4). The quadriceps muscle was tested through the extension of the hip in the prone position (Figure 5). The pelvis and the non-evaluated leg were fixed to the gurney with tapes, so as to avoid any type of displacement or compensation. The measurement was taken when the subject indicated that he could not withstand a greater tension or showed discomfort in any region. Measurements were taken three times consecutively and the highest value was chosen. A long-arm goniometer (Jamar) was used to obtain the degrees of elasticity. The same two evaluators (physiotherapists) conducted all the extensibility tests and always undertook the same roles. Based on the elasticity data, each subject’s intermediate angle of both coxofemoral joints was quantified, both for flexion and extension. With the mean angle for each direction, the unified angle common to the entire sample was calculated. The calculation of this unified angle was determined with the following formula: *URP_ANGLE_ = Flexion Hip angle − Extension Hip angle*. The last part of this first visit included the time required for the subjects to become familiar with the isokinetic dynamometer (Biodex System Pro 3). For this purpose, the subjects performed the number of repetitions of knee flexion and extension they considered appropriate to adapt to both the modalities and evaluation speeds.

The second visit took place one week after the first, and the evaluations were conducted at least 72 h after the matches. What is more, the sample subjects could not do any training during the 24 h preceding the tests. The isokinetic knee evaluation protocol was carried out according to the methodology of Croisier et al. [14,17]. The protocol included the performance of three repetitions at a speed of 60°/s following the concentric/concentric modality (H_CON60_/Q_CON60_), five repetitions at 240°/s according to those same modalities (H_CON240_/Q_CON240_), and three repetitions for the hamstrings, which were evaluated at 30°/s, under the eccentric and concentric modality (H_ECC30_/Q_CON30_) in both legs. During the isokinetic tests, verbal feedback was provided. However, during the execution, they were not allowed to view the isokinetic screen, where the graphs of muscle strength generation could be observed. One minute was used for both the rest between sets, as well as for switching between one leg and the other.

This test protocol was repeated for each subject in two positions: the CSP and the URP, with a 15-min break between them. The choice of the starting position was determined randomly for each subject.

To adjust the seat angle to the URP (URP_ANGLE_), the backrest inclination was modified, and the seat was moved in the anteroposterior axis until the hip was positioned at an angle of 44°, according to the results obtained with the formula URP_ANGLE_ = 75.7° − 31.9° (Table 1, see Elasticity) and considering that the supine position presents an angle of 0° (Figure 3). In all cases, an arc of movement of the knee was established between a 90° flexion and a −10° extension; that is to say, an 80° ROM. Gravity control was performed at a 40° knee flexion.

The immobilization and restraint of the subjects in the isokinetic seat followed the criteria recommended by Magnusson [21] to obtain a high PT. Thus, the thorax was stabilized with two crossed tapes, one horizontal over the pelvis and the other over the thigh of the leg to be evaluated. The non-evaluated leg was not stabilized, allowing possible muscle synergies.

Like the elasticity measurements, the fixation and follow-up of the isokinetic protocols were always carried out by the same physiotherapists.

### 2.4. Statistical Analyses

The data were analyzed by means of the statistical package SPSS Version 22.0 (IBM Corp., Armonk, NY, USA). For the descriptive statistical analysis of the results obtained from the isokinetic dynamometer ((Hamstring PT between both evaluated positions (H_ECC30_), and conventional (H_CON60_/Q_CON60_) and functional (H_EXC30_/Q_CON240_) ratios of the dominant and non-dominant legs, for both positions)), mean and standard deviations of the PT were used. The normality of the variables was analyzed using the Kolmogorov-Smirnov test (*p* < 0.05). The means of the hamstring eccentric PT data at 30°/s were compared between dominant and non-dominant legs data, for both positions, with the intention of looking for asymmetries (n = 20). The average PT between the two positions was also compared by adding the PT of both legs for one position (n = 40) and comparing it with the other position. Finally, the dominant and non-dominant legs were compared for the CSP and URP positions, both for the conventional and functional ratios (n = 20). In this comparison of means, a paired t-test was used for the variables that showed normality. For the variables that did not show normality, the Wilcoxon signed-rank test was applied by the analysis of the difference of the medians. In all the cases, a value of alpha *p* < 0.05 was considered statistically significant.

## 3. Results

Table 1 shows the elasticity data of the two major muscle groups associated with the knee (hamstrings and quadriceps), in the two studied positions (CSP and URP).

In addition to being used to determine the unified angle, elasticity allowed observing the degree of shortening of the subjects’ hamstring muscles. With regard to the hamstrings’ elasticity, the mean value was 75.7 ± 16.4°. Moreover, reported in Table 1 is the mean value of quadriceps elasticity, which was 31.9 ± 8.84°.

Regarding the eccentric hamstring PT at 30°/s (Table 1), no significant differences were observed between the dominant and non-dominant legs, neither in the CSP (*p* = 0.18) nor in the URP (*p* = 0.52).

In Table 1, it can also be observed that the comparison between the two positions (URP vs. CSP) during the PT of the eccentric extension of the knee at 30°/s performed by each leg (n = 40, that is 40 legs) showed no significant difference (*p* = 0.086).

In Table 2, only the dominant legs were considered. This data showed lower eccentric PT results in the URP than in the CSP (12 subjects), in contrast with the dominant legs that showed a PT with lower values in the CSP than in the URP (eight subjects). In both cases, the differences are significant.

Table 3 shows the results for the conventional and functional ratios of the dominant and non-dominant legs, in the two positions studied.

When comparing the mean values obtained in the conventional ratio (H_CON60_/Q_CON60_), no significant differences were found (*p* = 0.86).

Regarding the functional ratio (H_ECC30_/Q_CON240_), the mean values found were above 98% for both positions, which, according to Ayala et al. [19], is a value considered normal. There were no significant differences between the positions studied for this functional ratio (*p* = 0.97).

## 4. Discussion

In the first place, it is necessary to explain the reason for this new evaluation position. Finding a neutral [22] and functional [20,24] position for an open sport, such as soccer, is almost impossible. It is worth noting the similarity found between the URP calculated from the hamstring and quadriceps elasticity tests and the angle analyzed by 3D in the article by Sanchez et al. [25]. In this article, it is explained that during the kick, the hip angle where the highest angular velocity occurs coincides with 42.4° at the moment of impact. This can be explained by the fact that the muscles are adapted to repetitive actions, such as the kick. The URP represents the moment where the hamstring and quadriceps have similar muscle tension. By subtracting the degrees of the quadriceps elasticity from the degrees of the hamstring elasticity, not only do we achieve an agonist/antagonist muscle balance that can be considered neutral during the isokinetic test, but we also place these muscles in a position similar to those of the game settings.

The SLR test not only allowed the calculation of the URP, but also revealed a high number of subjects with poor hamstring extensibility (Table A1). This insufficient elasticity is an added risk of injury [26].

Although no significant difference was found in the comparison of the eccentric PT means of the hamstrings between the two positions studied, there seems to be a tendency in the URP protocol to develop a lower PT, especially in the dominant leg. Such results may be related to specific muscle loading patterns associated with high-level soccer sports [27], better ball control, or the way that young soccer players are trained [28]. It must also be taken into account that there is a greater tendency to shorten the hamstrings (Table 1). This fact, together with the CSP of the test, causes an increasing hamstring tension until knee extension is reached. This tension comes not only from the muscular component but also from passive structures (ligaments, tendons, and fasciae), which also generate an extra and free resistance (without energy consumption) to the isokinetic device, which will interpret it as eccentric force performed. In fact, a recent study by Tyler et al. [29], which used a stretched hamstring position, showed that 50% of the torque recorded comes from a combination of the mass of the leg and passive tension itself.

In CSP, the eccentric strength of the hamstrings and the resistance of the passive structures (ligaments, tendons, and fascia) cannot be separated. However, with the evaluation technique using the URP, the pretension of the musculature and the resistance of the passive structures are reduced, so the eccentric strength of the hamstrings can be mainly recorded.

Different studies have shown that the CSP is the one that produces the highest levels of PT, while the prone or supine positions show lower values [20,22,24,30]. In fact, some studies found significant differences in eccentric PT between different positions [22,31]. These positions (supine or prone positions) are very extreme, and, from our point of view, they are equally ill-adapted to the reality of sports, as is the seated position [30].

The URP does not seem to show great differences, perhaps because it is an intermediate position between evaluation techniques as extreme as the supine, prone, or sitting positions. This idea was already brought up by Guex et al. [24], when they pointed out that the force values in intermediate positions would be between those produced in extreme positions. Nonetheless, small changes in hip flexion can alter the PT production of the two muscle groups, due to changes in the muscle balance above the knee, i.e., the ratio [23,32].

In our data (Table A1), when considering the dominant legs that show lower eccentric PT values in the URP than in the CSP (12 subjects; subjects 1, 2, 4, 5, 6, 11, 12, 13, 14, 15, 17 and 18), the difference is significant (*p* = 0.001) (Table 2). Likewise, when we look at the dominant legs whose PT show higher values in the URP than in the CSP (8 subjects; subjects 3, 7, 8, 9, 10, 16, 19 and 20), the difference is also significant (*p* = 0.003) (Table 2). This would indicate that the change in position does have an impact on PT production. However, with regard to these last results, it should be noted that with the exception of one participant (subject 8), all the sample subjects presented a shortening of the hamstrings of the dominant leg below normal (80°) [18], and five of them presented hip flexion values below 70° (subjects 3, 7, 9, 19 and 20).

We consider that the muscle shortening of the hamstrings causes excess muscle tension, which allows the development of a high PT while sitting. Based on these results, we can propose, as has been done in other studies [20,31], that the position of the hip influences the eccentric PT values in knee extension. An example of this is the benefit of the CSP to the production of PT, which was already found by Bohannon et al. [30]. Furthermore, we should consider whether this eccentric force, smaller than we assumed, especially in the dominant leg, is sufficient to prevent joint muscle injuries. This is important since low PT values of the eccentric muscles are a risk factor for injury (such as anterior cruciate ligament, ACL, and hamstrings) [33].

Finally, and in relation to PT, it should be noted that contrary to what would be expected, the position of the hip does not have an influence on bilateral ratios or asymmetries (Table 1). This is so because when varying the position of the hip, the changes caused on the muscle length-tension of both the hamstrings and the quadriceps are found in both the dominant and non-dominant leg (Table 1).

Similarly, we consider it important to analyze values at the individual level. Thus, when looking at individual values of asymmetry in the CSP, we found that a significant number of sample subjects (subjects 1, 2, 4, 5, 6, 7, 8, 9, 10 and 16) showed values of 10% above those considered normal [5]. In the URP, a total of 12 subjects (subjects 3, 6, 8, 9, 10, 12, 13, 15, 16, 18, 19 and 20) recorded asymmetry values higher than the limit of normality (10%) [5] (Table A1).

For the conventional and functional ratios (Table 3), no significant differences were found between the two positions (URP vs. CSP). Even when analyzing the individual values of the conventional ratio, we found no differences either. Twenty-five legs were found to be at risk of injury as they showed values below the 60% established as normal values [10,11]. Therefore, only three subjects did not present muscular imbalances (subjects 2, 14 and 18). In the URP, the results showed 24 legs at risk, and only four subjects reported normal values (subjects 6, 10, 14 and 15). Nevertheless, considering the functional ratio individually and taking into account the abovementioned 98% normality criterion [19], we found five individuals at risk in the URP (subjects 3, 8, 9, 16 and 18), contrasting with only two in the CSP (subjects 9 and 16). Furthermore, if we consider the theoretical normality values [18] of 0.9 in the URP, we find two subjects with lower values (subjects, 9 and 16), while none in CSP (Table A2).

However, other studies have found significant differences between other positions [17,34,35]. This could be related to the fact that, with regard to the angle of the hip position, the URP would be between the sitting position and the supine or prone.

Among the studies that found differences between the ratios, it is worth mentioning the one carried out by Deighan et al. [20], which was similar to ours and in which significant differences were found regarding the functional ratio but not the conventional one. These authors placed the rugby players in a semi-recumbent position, at a 10° hip flexion, that characterizes the anteriorization of the trunk during the race [20]. Despite the similarities with our study, we consider that their position does not conform to the sporting reality of soccer or rugby, open activities that involve more actions (jumping, kicking, among other movements) than a simple one-way movement.

We think that besides possibly reflecting the position of the hip during the kick more accurately, the URP also seems to maintain a similar tension of the muscle groups involved. Therefore, the URP demonstrates to be likely to represent a functional position with a relevant ecological validity, which is scarcely true for the CSP [20].

The study carried out by Kellis [23] is the only one that, like ours, analyzed the H_EXC30_/Q_CON240_ ratio in different hip positions and did not find significant differences, although it did find a clear trend in their mean values. In our case, as mentioned above, we did find differences individually, since we observed a greater number of players at theoretical risk in the URP.

According to recent studies, an alteration in the H/Q ratio is caused by an elevated quadriceps PT [36]. However, we consider that the cause of an H/Q ratio with values below normal is rather by a low hamstring PT, the product of insufficient or no muscle compensation. Furthermore, we currently know that PT values vary, to a great extent, according to the position of the hip in which they are evaluated. For this reason, we think that finding more subjects with lower eccentric PT values in the URP position than in the CSP is normal.

Obviously, in training sessions and games, there is a disproportionate increase in the quadriceps strength on the hamstring. This is so because actions such as jumping or kicking involve the extension of the knee [36]. At the same time, we consider that the compensation for this alteration in the H/Q ratio should not come from the loss of quadriceps PT, but rather from the improvement of eccentric hamstring PT values through specific eccentric training [37]. This specific training could even be performed on the isokinetic device, using the URP, to allow a better transfer to real practice.

For all these reasons, we propose, as did Deighan, Kellis and Houtz [20,23,38], that the choice of the position for evaluating the isokinetic strength influences the results of the functional ratio. Additionally, this seems to be so when there is greater discrimination between patients and not when analyzing the sample as a whole. Based on the aforementioned studies [2,20,31], as well as on our data on the generation of a greater eccentric PT of the hamstrings and a smaller number of subjects with muscular imbalances in the CSP, we consider that the complementary use of the URP is of interest for the estimation of the individual risk of injury using the functional ratio.

Our study is not without its limitations. First, we are aware of the small sample used for this study. This is because we used the only team that plays competitively in the region, which limited our sample. In addition, enlarging the sample with subjects from other teams of lower sporting levels would have meant an alteration of the data, due to the subjects’ much lower physical condition than that of the subjects used. Second, it is worth mentioning the difficulty of placing subjects in the calculated hip position, due to the fact that the isokinetic dynamometer only has five intermediate positions between sitting and lying down. This was solved through the anteroposterior displacement of the seat and, to achieve lumbar support in these circumstances, specially designed cushions were used. Third, in this study, the protocol used was that of Croisier et al. [14,17], where the eccentric part is performed at the end. However, one of the possible limitations of our study may result from a certain degree of muscle fatigue on the evaluated limb itself, as well as on the non-evaluated one, from the crossover phenomenon; an effect that has been observed to be higher when training is based on eccentric contractions compared to the concentric modality [39].

Fourth, it is necessary to explain that most of the referenced studies that find significant differences between positions use extreme positions, such as the supine [17,34,35] or prone [22,35] position, which makes it easier to find differences in the ratio. Fourth, it must be considered that according to the studies developed by Alt et al. [40], the use of a maximum fastening system may have caused a decrease in force production. Despite the difficulty of finding the intermediate positions used in our study, we believe that these data provide greater utility, since they were obtained in a position of agonist-antagonist muscle balance, which is also closer to the positions developed by the hip during matches. Therefore, we find it interesting to combine our results with those of other authors [9], since they seem to be the best approximations of a functional isokinetic evaluation. Finally, it is worth mentioning that this manuscript is the result of a pilot study serving as the basis for a larger one. The next experimental phase will be to use an individualized hip angle for each subject, for greater precision in the tensional balance of the thigh musculature during isokinetic execution.

## 5. Conclusions

The absence of significant results does not allow us to confirm the great utility that the URP may have in the assessment of these imbalances. Nonetheless, the detection of a greater number of subjects at theoretical risk of injury favors the idea that new studies could suggest the inclusion of this protocol in the isokinetic assessment of the knee.

The dominant leg is usually the one that presents a greater shortening at the hamstring level and a greater difference in the eccentric PT data. This seems to indicate that muscle shortening generates greater tension during the test in the CSP position, originating higher results than in URP. The tension produced in passive structures (ligaments, tendons and fasciae) seems to be responsible for a greater eccentric hamstring PT in the CSP position.

The CSP and the URP could complement each other to improve the anticipation of a possible injury, facilitating its prevention in any individual, whether an athlete or not. Moreover, isokinetic training in this position could strengthen the thigh muscles concentrically and eccentrically, allowing better transfer to actual practice. Finally, this functional position could also enable the monitoring of the rehabilitation process and ensure the reintroduction of the player to training and/or competition with a lower risk of recidivism. In the meantime, it would be of interest to foment studies with this new experimental position or with others that could improve injury prediction.

## Figures and Tables

**Table 1:** Condition of the hamstring and quadriceps musculature: elasticity and eccentric PT in a 30°/s knee extension (H_EXC30_). The data for each group (n = 20) are reported as mean and standard deviation (mean ± SD).

Hamstring Elasticity (°)		Hamstring Peak Torque					
		Classic Sitting Position (CSP)			Unified Reclined Position (URP)		
Dom	N-Dom	H_EXC30_ (Nm)		Asymmetry (%)	H_EXC30_ (Nm)		Asymmetry (%)
74.1 ± 14.9	77.4 ± 18	**Dom leg**	**N-Dom leg**	**ER Dom/N-Dom**	**Dom leg**	**N-Dom leg**	**ER Dom/N-Dom**
75.7 ± 16.4 *		152 ± 22.4	144 ± 16	11.9 ± 8.33 *	144.9 ± 22.8	142 ± 24.2	12.1 ± 8.73 *
Quadriceps elasticity: 31.9 ± 8.84 **		*p*-value = 0.18 (n.s.)			*p*-value = 0.52 (n.s.)		
URP = 75.7° − 31.9°		**Dom + N-Dom (n = 40)**: 148.3 ± 19.5			**Dom + N-Dom (n = 40)**: 143.5 ± 23.2		
URP = 43.8° ≈ 44°		*p*-value = 0.086 (n.s.)					

NOTE: the asymmetry values represented in light gray express the superiority of the dominant leg over the non-dominant one, while values represented in dark gray indicate that the non-dominant leg presents higher values when compared to the dominant one. Dom = dominant; N-Dom = non dominant. * values considered not normal when compared to literature. ** Quadriceps elasticity angle obtained with the extensibility test in the prone position. Dom—dominant; Non-Dom—non-dominant. n.s.—not significant.

**Table 2:** Comparison of means of the subjects reporting lower values of eccentric hamstring PT (H_EXC30_) in URP versus CSP (URP < CSP), and vice versa (CSP < URP).

Dominant Leg: Eccentric Hamstring Peak Torque 30°/s (H_exc30_)			
URP < CSP (n = 12)		CSP < URP (n = 8)	
CSP: 156.4 ± 24.3	URP: 139.6 ± 23	CSP: 144.3 ± 18.4	URP: 153 ± 21.3
*p* = 0.001		*p* = 0.003	

**Table 3:** Conventional (H_CON60_/Q_CON60_) and functional (H_EXC30_/Q_CON240_) ratios in CSP and URP. Data for each group (n = 20) are shown as mean and standard deviation (mean ± SD).

Conventional Ratio (H_CON60_/Q_CON60_)				Functional Ratio (H_EXC30_/Q_CON240_)			
Classic Sitting Position (CSP)		Unified Reclined Position (URP)		Classic Sitting Position (CSP)		Unified Reclined Position (URP)	
Dom leg	N-Dom leg	Dom leg	N-Dom leg	Dom leg	N-Dom leg	Dom leg	N-Dom leg
60.5 ± 7.07	61.9 ± 20.6	61.0 ± 8.99	62.3 ± 14	130 ± 24.9	129 ± 25.8	128 ± 20.6	130 ± 39
61.2 ± 13.8		61.7 ± 11.5		129.5 ± 25.4		129 ± 29.8	
*p* value = 0.86 (n.s.)				*p* value = 0.97 (n.s.)			

Values expressed in percentages. Dom = dominant; N-Dom = non dominant. n.s.—not significant.

## Data Availability

All data generated or analyzed during this study are included in this published article.

## Appendix A

**Table A1:** Individual values of the hamstring muscles elasticity and eccentric PT in knee extension at 30°/s (Hexc30).

Subjects	Hamstring Elasticity (°)		Hamstring Peak Torque					
			Classic Sitting Position (CSP)			Unified Reclined Position URP)		
	Dom	N-Dom	H_EXC30_ (Nm)		Asym (%)	H_EXC30_ (Nm)		Asym (%)
1	82	84	162.3	141.8	12.7 *	159.7	166.2	4
2	69 *	69 *	163.7	140.7	14 *	151	139.4	7.7
3	52 *	60 *	141.6	142.1	4	153.7	122.7	20.2 *
4	84	72 *	156.7	138.7	11.5 *	142.6	151	5.9
5	82	114	203.6	160.7	21 *	171.5	178.8	4.3
6	105	119	166.8	132.4	20.6 *	128.5	109.9	14.5 *
7	60 *	65 *	140.3	160.3	14.2 *	140.9	140.3	0.4
8	90	100	147.9	181.7	22.8 *	168	186.5	11 *
9	73 *	77 *	153.4	126.4	17.6 *	160	122.1	23.7 *
10	78 *	64 *	169.6	147.3	13.1 *	185.3	155.4	16.2 *
11	90	80	127.8	128.7	0.7	114.7	111.4	2.9
12	54 *	60 *	134.2	124.8	7	126.5	113	10.7 *
13	65 *	78 *	150.1	136.9	8.8	137.2	155.6	13.4 *
14	78 *	80	193.1	177.9	7.8	175.8	172.5	1.8
15	95	85	125.1	135.7	8.4	100.1	133.2	33 *
16	68 *	78 *	112.4	151.4	34.6 *	120.6	96.8	19.7 *
17	78 *	89	156.1	152.9	2	148	145.6	1.6
18	50 *	50 *	137.1	146.7	7	119.3	145.1	21.6 *
19	69 *	60 *	161.8	147.5	8.9	165	143.7	12.9 *
20	60 *	63 *	127.3	124.9	1.9	130.4	150.7	15.6 *

NOTE: the asymmetry values represented in light gray express the superiority of the dominant leg over the non-dominant one, while values represented in dark gray indicate that the non-dominant leg presents higher values when compared to the dominant one. Dom = dominant; N-Dom = non dominant; Asym = asymmetry. * values considered not normal when compared to literature.

**Table A2:** Individual values of conventional (H_CON60_/Q_CON60_) and functional (H_EXC30_/Q_CON240_) ratio in the classic sitting position (CSP) and in the unified reclined position (URP).

Subjects	Conventional Ratio (H_CON60_/Q_CON60_)				Functional Ratio (H_EXC30_/Q_CON240_)			
	Classic sitting Position (CSP)		Unified Reclined Position (URP)		Classic Sitting Position (CSP)		Unified Reclined Position (URP)	
	Dom Leg	N-Dom Leg	Dom Leg	N-Dom Leg	Dom Leg	N-Dom Leg	Dom Leg	N-Dom Leg
1	57.7 *	52.9 *	47.7 *	47.4 *	128.4	113.5	123.2	120.0
2	72.8	62.1	77.2	54.5 *	161.0	137.0	172.0	114.0
3	64.2	56.6 *	56.3 *	55.6 *	114.1	114.0	118.1	88.0 **
4	62.8	50.9 *	63.7	57.2 *	121.0	108.2	119.4	107.9
5	58.2 *	53.2 *	58.8 *	59.8 *	125.2	104.8	112.6	121.6
6	50.8 *	51.6 *	78.7	69.2	191.9	138.5	150.6	110.6
7	60.3	53.9 *	56.9 *	61.7	138.1	161.9	125.2	137.8
8	60.6	52.2 *	55.1 *	59.1 *	125.0	116.7	95.6 **	129.2
9	51.4 *	43.9 *	55.2 *	48.6 *	104.4	94.9 **	120.1	84.9 **
10	65.0	57.2 *	67.3	62.3	116.0	110.2	123.9	108.1
11	55.6 *	59.7 *	59.3 *	53.1 *	134.0	148.1	143.2	150.5
12	57.4 *	42.1 *	62.3	51.1 *	119.2	109.7	100.7	106.0
13	61.9	59.8 *	51.7 *	56.8 *	159.7	155.2	189.8	203.9
14	66.9	124.9	69.0	110.4	170.4	206.9	174.1	230.0
15	59.3 *	95.9	64.1	82.2	124.0	144.2	120.9	179.5
16	52.0 *	57.7 *	54.5 *	61.7	94.5 **	131.4	98.5	88.2 **
17	62.3	57.3 *	56.3 *	58.9 *	146.2	129.7	140.7	123.9
18	79.2	97.9	77.9	77.5	99.3	117.6	95.0 **	119.8
19	58.1 *	46.3 *	57.7 *	53.4 *	111.4	116.4	110.6	109.7
20	52.7*	61.5	51.1 *	66.3	115.9	113.6	122.9	176.3

Values expressed in percentages. Dom = dominant; N-Dom = non dominant. * According to literature, these results refer to values below normal for the conventional ratio; ** According to literature, these results refer to subjects at risk of injury, considering the functional ratio.
